# Lymph node metastasis-derived gastric cancer cells educate bone marrow-derived mesenchymal stem cells via YAP signaling activation by exosomal Wnt5a

**DOI:** 10.1038/s41388-021-01722-8

**Published:** 2021-03-02

**Authors:** Mei Wang, Xinxin Zhao, Rong Qiu, Zheng Gong, Feng Huang, Wanjun Yu, Bo Shen, Xin Sha, Haibo Dong, Jiaying Huang, Lin Wang, Wei Zhu, Wenrong Xu

**Affiliations:** 1 Key Laboratory of Medical Science and Laboratory Medicine of Jiangsu Province, School of Medicine, Jiangsu University, Zhenjiang, Jiangsu Province China; 2 Department of Oncology, Jiangsu Cancer Hospital & Jiangsu Institute of Cancer Research, Nanjing Medical University Affiliated Cancer Hospital, Nanjing, Jiangsu Province China; 3 Department of Surgery, The Affiliated Hospital of Jiangsu University, Jiangsu University, Zhenjiang, Jiangsu Province China; 4 Department of Hematology, Nanjing Drum Tower Hospital, The Affiliated Hospital of Jiangsu University, Nanjing, Jiangsu Province China

**Keywords:** Cancer microenvironment, Gastric cancer

## Abstract

Lymph node metastasis (LNM), a common metastatic gastric-cancer (GC) route, is closely related to poor prognosis in GC patients. Bone marrow-derived mesenchymal stem cells (BM-MSCs) preferentially engraft at metastatic lesions. Whether BM-MSCs are specifically reprogrammed by LNM-derived GC cells (LNM-GCs) and incorporated into metastatic LN microenvironment to prompt GC malignant progression remains unknown. Herein, we found that LNM-GCs specifically educated BM-MSCs via secretory exosomes. Exosomal Wnt5a was identified as key protein mediating LNM-GCs education of BM-MSCs, which was verified by analysis of serum exosomes collected from GC patients with LNM. Wnt5a-enriched exosomes induced YAP dephosphorylation in BM-MSCs, whereas Wnt5a-deficient exosomes exerted the opposite effect. Inhibition of YAP signaling by verteporfin blocked LNM-GC exosome- and serum exosome-mediated reprogramming in BM-MSCs. Analysis of MSC-like cells obtained from metastatic LN tissues of GC patients (GLN-MSCs) confirmed that BM-MSCs incorporated into metastatic LN microenvironment, and that YAP activation participated in maintaining their tumor-promoting phenotype and function. Collectively, our results show that LNM-GCs specifically educated BM-MSCs via exosomal Wnt5a-elicited activation of YAP signaling. This study provides new insights into the mechanisms of LNM in GC and BM-MSC reprogramming, and will provide potential therapeutic targets and detection indicators for GC patients with LNM.

## Introduction

Gastric cancer (GC), a common gastrointestinal malignancy, shows a high morbidity and mortality in China, and is a burden on public health [1]. Although there are multiple therapeutic strategies available, the prognosis of patients diagnosed at advanced stage remains very poor [2]. Regional lymph node metastasis (LNM), an independent risk factor for prognosis in GC patients [3], is an initial step in cancer-cell dissemination and metastasis throughout the body [4, 5]. Regional LNM plays a crucial gateway role in cancer spread, but the underlying mechanism of LNM remains unclear.

Bone marrow-derived mesenchymal stem cells (BM-MSCs) exhibit strong tropism to inflammatory lesions and serve as an important cellular origin for tumor stromal cells [6]. Most current studies have investigated the recruitment of BM-MSCs into primary tumors, the reprogramming of BM-MSCs into various tumor stromal cells, and tumor-promoting effects of BM-MSCs [7]. However, whether BM-MSCs are incorporated into metastatic lesions remains unclear. Xie et al. found that the circulation time of BM-MSCs in mice with metastatic lung cancer was brief, and that BM-MSCs displayed increased metastatic tissue tropism [8]. Additionally, only cancer cells with higher metastatic capacity can trigger BM-MSC transition into-tumor-associated stromal cells [9–11]. Metastasis-associated MSCs are found in the metastatic LN and liver tissues of breast-cancer patients [12]. These findings suggest that BM-MSCs preferentially engraft at metastatic sites, and are educated by metastatic cancer cells to incorporate into the metastatic microenvironment. The recruitment of MSCs in primary GC is closely related to LNM [13]. However, whether BM-MSCs are specifically educated and directly incorporated into the LN microenvironment to promote the malignant progression of GC after LNM remains unknown.

In this study, we first compared metastatic LN-derived GC cells (LNM-GCs) and primary GC-derived cancer cells (primary GCs) in their ability to educate BM-MSCs. Then, we investigated the critical paracrine and molecular factors participating in LNM-GC-mediated education of BM-MSCs. Finally, we examined the downstream signaling involved in this process. MSC-like cells derived from regional LNM and serum samples of GC patients with and without regional LNM were also included in our analysis.

## Results

### Paracrine effects of LNM-GC-mediated education of BM-MSCs are regulated by secretory exosomes

**Fig. 1:**
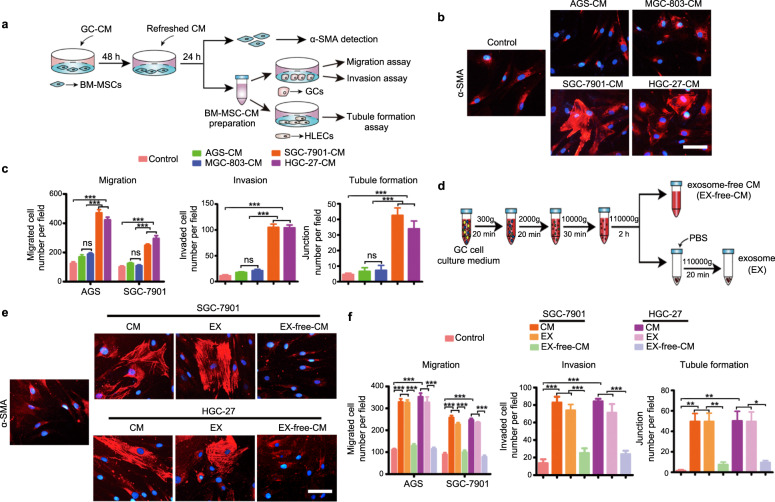
Exosomes are critical paracrine contributors to LNM-GC mediated education of BM-MSCs. **a** The flow chart illustrates the process of treating BM-MSCs with GC-CM, and evaluation of the resulting BM-MSC phenotype and function. **b**, **c** The ability to educate BM-MSCs was compared between LNM-GCs (SGC-7901 and HGC-27) and primary GCs (AGS and MGC-803). **d** LNM-GC exosomes and exosome-free CM were prepared as indicated in the flow chart. **e**, **f** BM-MSCs were treated with CM, exosomes, or exosome-free CM from LNM-GCs. The educational effect of the three treatments on BM-MSCs was then compared. **b**, **e** Immunofluorescence detection of α-SMA in BM-MSCs was conducted to detect their tumor-promoting phenotype (magnification, ×200; scale bars, 50 μm). **c**, **f** GC migration and invasiveness analysis, and HLEC tubule formation assay, were performed to analyze the tumor-promoting function of BM-MSCs. The number of migrated and invaded GCs and tubule junctions were counted and represented in column charts. Data are presented as the mean ± SD of three independent experiments. Statistical significance was calculated using one-way analysis of variance (ANOVA) followed by Tukey’s test. ***P < 0.0001; **P < 0.01; *P < 0.05; ns, non significant.

Conditioned media (CM) from primary GCs (AGS and MGC-803) and LNM-GCs (SGC-7901 and HGC-27), to be used for the treatment of BM-MSCs, were prepared separately. The effects of CM-mediated education of BM-MSCs were evaluated as indicated in the flow diagram shown in Fig. 1a. Compared with those of the control, the expression levels of alpha-smooth muscle actin (α-SMA), a typical marker of tumor-associated stromal cells, were increased in BM-MSCs treated with SGC-7901-CM or HGC-27-CM. However, the levels of α-SMA were unaltered in BM-MSCs cultured in AGS-CM or in MGC-803-CM (Fig. 1b). CM derived from BM-MSCs treated with HGC-27-CM or SGC-7901-CM markedly promoted AGS and SGC-7901 migration, HGC-27 invasion, and lymphatic capillary endothelial cell (HLEC) tubule formation; however, CM from BM-MSCs cultured in AGS-CM or MGC-803-CM did not exert these effects (Fig. 1c). These results suggest that LNM-GCs educated BM-MSCs by endowing them with tumor-promoting phenotype and function via a paracrine route.

To explore the critical factors involved in LNM-GC CM-mediated education of BM-MSCs, an exosome fraction (pellet) and an exosome-free fraction (supernatant) were separately prepared from SGC-7901-CM and HGC-27-CM using differential ultracentrifugation as shown in Fig. 1d. Exosomes and exosome-free CM from an equal volume of LNM-GC CM were used to treat BM-MSCs. As shown, particles with exosome-like size and specific marker expression were detected in GC-CM. However, particles with exosomal characteristics were not observed in exosome-free-CM (Supplementary Fig. 1a–c). Treatment with exosomes significantly induced α-SMA expression in BM-MSCs compared with the levels of the controls. Treatment with exosomes also enhanced the ability of BM-MSCs to promote GC migration, invasion, and HLEC tubule formation. The effects of this treatment were similar to those exerted by LNM-GC CM. However, removal of exosomes eliminated the educational effects of LNM-GC CM on BM-MSCs (Fig. 1e, f). These results indicate that exosomes are crucial paracrine contributors to LNM-GC-mediated education of BM-MSCs.

### LNM-GC exosomes possess an enhanced capability to reprogram BM-MSCs into cancer-associated MSCs

**Fig. 2:**
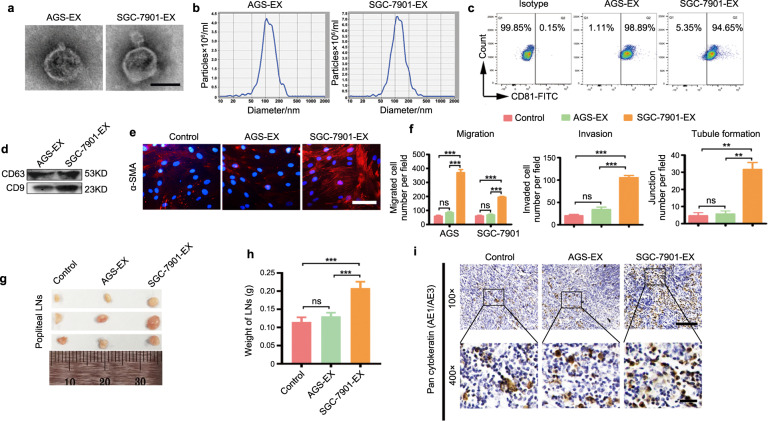
LNM-GC-derived exosomes exhibit an enhanced ability to educate BM-MSCs relative to the ability of primary GC-exosomes. **a**–**d** Characterization of AGS- and SGC-7901-exosomes. **a** Representative transmission electron microscopy (TEM) images (magnification, ×60,000; scale bars, 100 μm). **b** Size distribution shown by nanoparticle tracking analysis (NTA). **c** Flow cytometry analysis of CD81 expression. **d** Western blotting analysis of CD63 and CD9 expression. **e**–**i** The ability of AGS- and SGC-7901-exosomes to educate BM-MSCs was evaluated. **e** α-SMA expression in BM-MSCs (magnification, ×200; scale bars, 50 μm). **f** Quantification of migration, invasion, and tubule formation. **g**–**i** In vivo analysis of tumor-promoting properties of BM-MSCs. **g** The draining popliteal lymph nodes (LNs) from each group were harvested and imaged. **h** Weight of popliteal LNs. **i** Immunohistochemistry analysis of pan-cytokeratin (AE1/AE3) expression in popliteal LNs. Representative images from each group are shown (magnification, ×100, scale bars, 100 μm; magnification, ×400, scale bars, 20 μm). Data are expressed as the mean ± SD of three independent experiments. Statistical significance was calculated using one-way ANOVA followed by Tukey’s test. ***P < 0.0001; **P < 0.01; ns, non significant.

To confirm the critical role of exosomes in LNM-GC-mediated education of BM-MSCs, AGS and SGC-7901 were separately selected to represent primary GCs and LNM-GCs, and their respective exosomes were isolated by differential ultracentrifugation. These isolated exosomes displayed a lipid-bilayer membrane structure and typical exosomal sizes, and expressed the exosomal markers CD81, CD63, and CD9 (Fig. 2a–d). These exosomes were then used to treat BM-MSCs. Our results show that increased α-SMA expression occurred only in BM-MSCs treated with SGC-7901 exosomes (Fig. 2e). The in vitro functional analysis showed that the migratory and invasive capacities of GCs, as well as tubule-forming ability of HLECs, were markedly enhanced by CM from BM-MSCs treated with SGC-7901 exosomes (Fig. 2f). In vivo, SGC-7901 pretreated with BM-MSC-CM was used to establish a mouse model of LNM. The results showed that the volume and weight of popliteal LNs in the group of BM-MSCs treated with SGC-7901 exosomes were notably larger and heavier than those in the other two groups (Fig. 2g, h). Pan-cytokeratin AE1 and AE3 labeling showed that micrometastases in the group of BM-MSCs treated with SGC-7901 exosomes were more apparent than those in the other two groups (Fig. 2i). However, no differences were observed between the group of BM-MSCs treated with AGS exosomes and the control group. Similar results were observed in the AGS tumor model. In this model, metastases occurred only in the group of BM-MSCs treated with SGC-7901 exosomes (Supplementary Fig. 2). These results suggest that LNM-GC exosomes showed an enhanced capability to educate BM-MSCs, further validating the notion that exosomes play a critical role in this process and indicating that LNM-GC-educated BM-MSCs enable primary GCs to acquire the ability to metastasize to LNs.

### Exosome protein cargo is critical for LNM-GC-mediated education of BM-MSCs

**Fig. 3:**
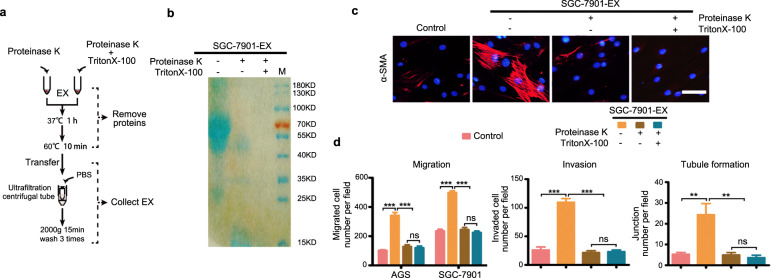
Removal of proteins from SGC-7901 exosomes eliminates their participation in education of BM-MSCs. **a** The flow chart shows removal of exosomal proteins by proteinase K alone or combined with Triton X-100, and purification of these treated exosomes by ultrafiltration centrifugal tubes. **b** SDS-PAGE and Coomassie brilliant blue stain were used to confirm that proteins were indeed removed from SGC-7901 exosomes. **c**, **d** The influence of protein removal on education of BM-MSCs by SGC-7901 exosomes. **c** Immunofluorescence detection of α-SMA expression in BM-MSCs (magnification, ×200; scale bars, 50 μm). **d** Quantification of migrated and invaded GCs, and formed tubule junctions. Data are presented as the mean ± SD of three independent experiments. Statistical significance was assessed using one-way ANOVA followed by Tukey’s test. ***P < 0.0001; **P < 0.01; ns, non significant.

Metastatic cancer cell-exosomes show a distinct metastasis-associated protein profile [14–16]. To investigate whether the protein cargo loaded in LNM-GC exosomes was involved in education of BM-MSCs, SGC-7901-derived exosomes were pretreated with proteinase K alone or combined with Triton X-100 to remove the surface proteins or the entire protein cargo, respectively (Fig. 3a, b), and were then used to treat BM-MSCs. Our results show that both types of these pretreated exosomes lost their ability to induce α-SMA expression in BM-MSCs (Fig. 3c), or to endow BM-MSCs with capability to promote GC migration and invasion, and stimulation of HLEC tubule formation (Fig. 3d). No obvious differences were observed between the two pretreated groups of exosomes. Similar results were observed in HGC-27 exosomes (Supplementary Fig. 3). These findings suggest that surface proteins of LNM-GC exosomes are vital in exosome-mediated education of BM-MSCs.

### Exosome-delivered Wnt5a modulates LNM-GC-mediated reprogramming of BM-MSCs

**Fig. 4:**
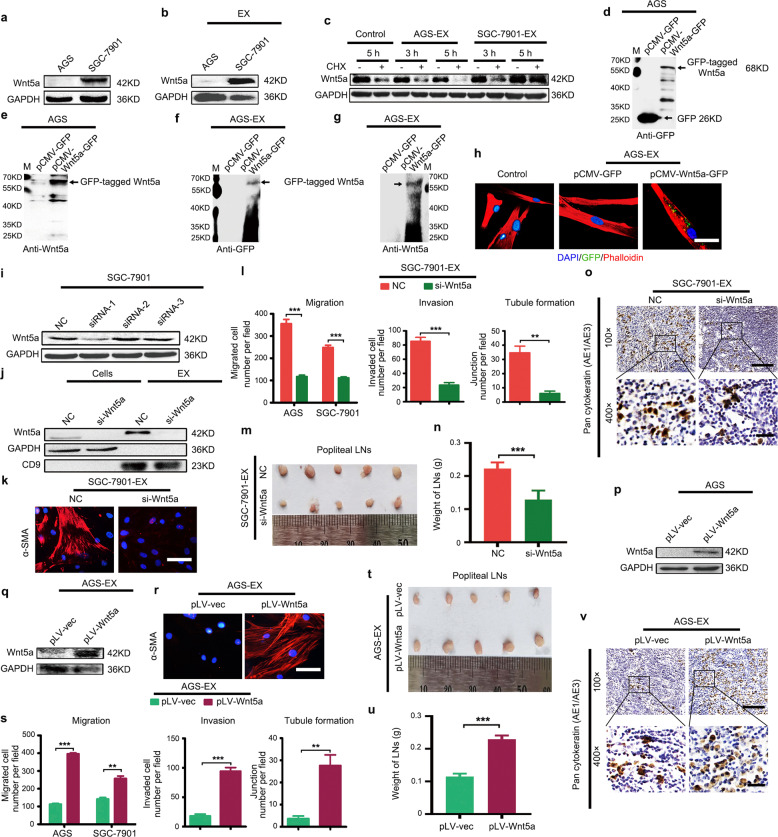
Exosomal Wnt5a regulates LNM-GC-mediated reprogramming of BM-MSCs. **a**, **b** Comparison of cellular and exosomal levels of the Wnt5a protein in AGS and SGC-7901 using western blotting. **c** Detection of Wnt5a protein in BM-MSCs after treatment with CHX (15 μg/ml) alone or with exosomes at the indicated times. **d**–**g** AGS stably transfected with pCMV-GFP and pCMV-Wnt5a-GFP were separately established. GFP and Wnt5a in cells and their exosomes were individually detected by western blot. **h** Exosome internalization analysis in BM-MSCs after incubation with exosomes from the abovenamed two stably transfected AGS (magnification, ×400; scale bars, 25 μm). **i** Screening for the most efficient siRNA against Wnt5a (si-Wnt5a) in SGC-7901. **j** Western blotting analysis of Wnt5a expression in SGC-7901 cells, and analysis of their secreted exosomes after transfection of SGC-7901 with si-Wnt5a and NC. **k**–**o** si-Wnt5a- and NC-transfected SGC-7901 exosomes were separately used to treat BM-MSCs in order to compare their effects in education of BM-MSCs. **p**, **q** Western blotting analysis of Wnt5a in AGS and in their secreted exosomes after AGS were infected with the pLV-Wnt5a lentivirus. **r**–**v** Exosomes derived from pLV-Wnt5a- and pLV-vec-lentivirus-infected AGS were separately used to treat BM-MSCs to observe their BM-MSC-reprogramming abilities. **k**, **r** Detection of α-SMA expression in BM-MSCs (magnification, ×200; scale bars, 50 μm). **l**, **s** Numbers of migrated and invaded GCs and formed tubule junctions. **m**–**o**, **t**–**v** In vivo analysis of tumor-promoting properties in BM-MSCs. **m**, **t** The draining popliteal LNs harvested from the above groups. **n**, **u** Weight of popliteal LNs. **o**, **v** Pan-cytokeratin (AE1/AE3) expression in popliteal LNs detected using immunohistochemistry (magnification, ×100, scale bars, 100 μm; magnification, ×400, scale bars, 20 μm). Data are presented as the mean ± SD of three independent experiments. Statistical significance was assessed using Student’s *t* test. ***P < 0.0001; **P < 0.01.

To determine which proteins were critical in BM-MSC reprogramming, we paid attention to the proteins that have been shown to be present on the exosomal surface, and we found the activated Wnt proteins [17, 18]. A recent meta-analysis also revealed that positive expression of Wnt5a is correlated with LNM in GC [19]. Expectedly, Western blotting showed that the expression levels of Wnt5a were significantly elevated in LNM-GCs and also highly enriched in LNM-GC exosomes (Fig. 4a, b and Supplementary Fig. 4a, b). To determine whether Wnt5a could be delivered into BM-MSCs through exosomes, we used the protein synthesis inhibitor cycloheximide (CHX) to treat BM-MSCs. We found that its endogenous levels were dropped quickly after treatment for 3 and 5 h (Supplementary Fig. 4c). When BM-MSCs were treated with CHX and exosomes simultaneously, Wnt5a protein levels were not lower but significantly higher in the SGC-7901 exosome group than in the other groups (Fig. 4c). Furthermore, we established an AGS cell line stably expressing GFP-tagged Wnt5a (pCMV-Wnt5a-GFP). The corresponding vector (pCMV-GFP) was served as a control (Fig. 4d, e). Exosomes were separately isolated from the stably transfected AGS (Supplementary Fig. 1d, e). GFP-tagged Wnt5a was detectable in pCMV-Wnt5a-GFP-AGS-derived exosomes, but neither GFP nor Wnt5a was tested in the control group (Fig. 4f, g). The internalization assay showed that small green fluorescent particles were only observed in cytoplasm of BM-MSCs treated with pCMV-Wnt5a-GFP-AGS exosomes (Fig. 4h). These results establish that Wnt5a is specifically loaded in exosomes and is directly transferred into BM-MSCs. Therefore, we selected Wnt5a for further investigation.

To explore whether exosomal Wnt5a was involved in LNM-GC-mediated education of BM-MSCs, three pairs of short interfering RNA fragments (siRNAs) against Wnt5a were synthesized to suppress Wnt5a expression in SGC-7901 cells (Supplementary Table 1, Fig. 4i). Western blotting showed that knockdown of Wnt5a in SGC-7901 cells resulted in reduced exosomal content of Wnt5a (Fig. 4j). The siRNAs with the highest transfection efficiency were used as si-Wnt5a in consequent experiments. Exosomes were separately isolated from NC- and si-Wnt5a-transfected SGC-7901 cells, and were then used to treat BM-MSCs. Compared with the expression levels of NC group, α-SMA expression was nearly undetectable in the si-Wnt5a group (Fig. 4k). CM, prepared from BM-MSCs that had been treated with Wnt5a-deficient exosomes, could not promote GC migration and invasion or HLEC tubule formation (Fig. 4l). Similar results were obtained in HGC-27 cells (Supplementary Fig. 4d–g). Additionally, smaller volume and decreased weight of popliteal LNs and reduction in areas positive for pan-cytokeratin (AE1 and AE3) in popliteal LNs were observed in si-Wnt5a-treated group compared with those of the NC group in vivo (Fig. 4m–o).

To further validate the notion that GC exosomes educated BM-MSCs using Wnt5a signaling, we overexpressed Wnt5a in AGS cells using lentivirus infection (pLV-Wnt5a) and obtained Wnt5a-enriched AGS exosomes (Fig. 4p, q). Compared with the pLV-Vec group, Wnt5a-enriched exosomes induced significantly increased expression of α-SMA in BM-MSCs, and enhanced BM-MSC-mediated promotion of GC migration and invasion, and HLEC tubule formation, in vitro (Fig. 4r, s). In vivo, we observed larger volumes, increased weight and increased areas positive for pan-cytokeratin (AE1 and AE3) in popliteal LNs of the pLV-Wnt5a group relative to those of the pLV-Vec group (Fig. 4t–v). Collectively, these results suggest that LNM-GCs induce BM-MSCs reprogramming via exosomal delivery of Wnt5a.

### Exosomes derived from the serum of GC patients with regional LNM can educate BM-MSCs, while Wnt5a content in serum exosomes is positively correlated with LNM

**Fig. 5:**
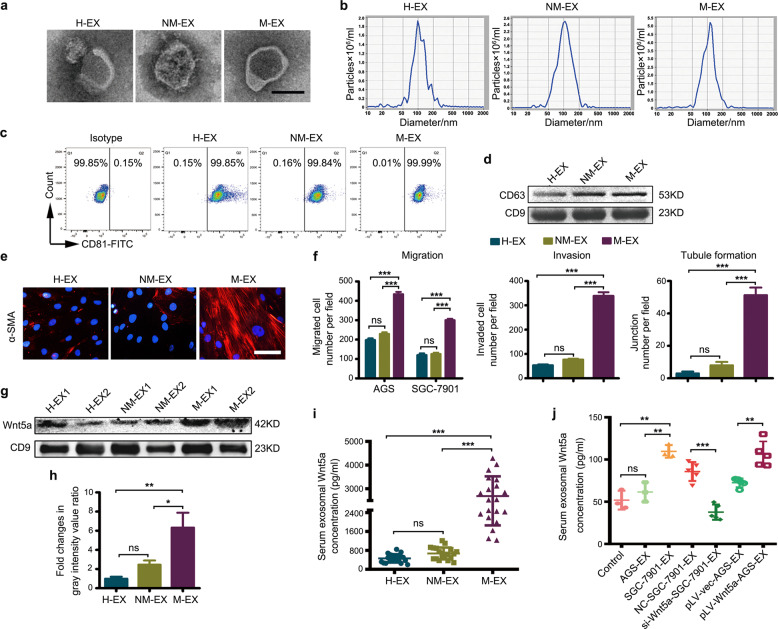
Serum exosomes, isolated from LNM-GC patients educate BM-MSCs, and circulating exosomal Wnt5a content is associated with LNM. **a**–**d** Characterization of serum exosomes isolated from healthy participants (H), GC patients without LNM (NM), and GC patients with LNM (M). **a** Representative TEM images of exosomes (magnification, ×60,000; scale bars, 100 μm). **b** Size distribution of exosomes analyzed using NTA; **c** Detection of CD81 expression by flow cytometry. **d** Western blotting analysis of CD63 and CD9 expression. **e**, **f** Comparison of BM-MSC-educating capacity among exosomes from the three groups described above. **e** Detection α-SMA expression in BM-MSCs (magnification, ×200; scale bars, 50 μm). **f** Analysis of tumor-promoting properties of BM-MSCs. **g**, **h** Wnt5a expression in exosomes isolated from the pooled sera was detected by western blotting. **g** Representative image. **h** Fold changes in relative gray values of Wnt5a; **i**, **j** Serum concentration of exosomal Wnt5a was detected using ELISA. **i** Serum concentration of exosomal Wnt5a in clinical serum samples (20 individuals per group). **j** Wnt5a concentration in serum samples from the LNM model described in Figs. 2, 4. Data are presented using means ± SD of three independent experiments. Statistical significance was determined using one-way ANOVA followed by Tukey’s test. ***P < 0.0001; **P < 0.01; *P < 0.05; ns, non significant.

Next, we separately purified serum exosomes obtained from healthy individuals (H), GC patients without (NM), and those with regional LNM (M). These exosomes exhibited typical exosomal morphological characteristics, size, and expression of specific protein markers (Fig. 5a–d). Sera obtained, from five patients per group, were pooled for exosome isolation. Our results demonstrate that exosomes derived from the M group showed increased a-SMA expression and enhanced tumor-promoting capacity in BM-MSCs (Fig. 5e, f). However, exosomes obtained from the other two groups did not exert these effects. These findings suggest that serum exosomes derived from GC patients with regional LNM harbored the ability to educate BM-MSCs.

To determine the Wnt5a expression profile in serum exosomes, we evaluated Wnt5a expression in the pooled serum exosomes using western blotting. Our results show that Wnt5a was highly enriched in the exosomes of the M group, but its content was very low in, and showed no differences between, the other two groups (Fig. 5g, h). Furthermore, exosomes, obtained from each participant in the three abovementioned groups, were isolated for Wnt5a detection using ELISA. Consistently, Wnt5a was highly expressed in the exosomes of the M group compared with those of the other two groups (Fig. 5i). Moreover, detection of serum exosomal Wnt5a in the LNM model showed that Wnt5a concentration was markedly increased in SGC-7901 and pLV-Wnt5a exosome groups, but reduced in the si-Wnt5a exosome group, compared with the respective control groups (Fig. 5j). Combined with the results previously obtained in mice, these findings suggest that Wnt5a was selectively sorted into serum exosomes derived from LNM-associated GCs, and was positively correlated with LNM. These findings provide novel insights into the BM-MSC-educating ability of serum exosomes isolated from GC patients with LNM.

### YAP activation, induced by exosomal Wnt5a, is pivotal for LNM-GC-mediated reprogramming of BM-MSCs

**Fig. 6:**
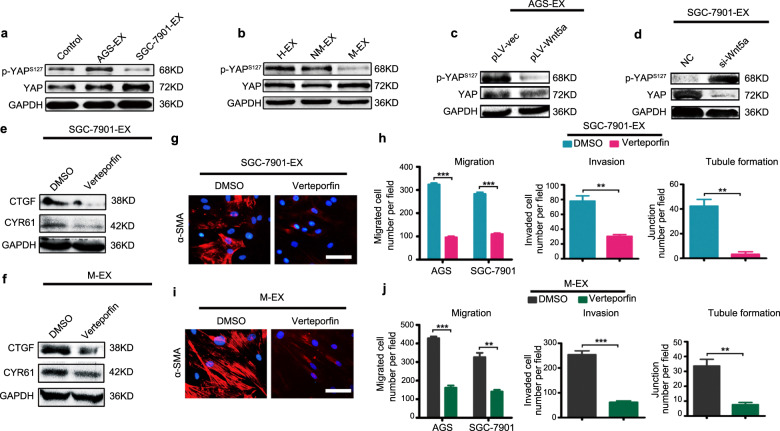
Exosomal Wnt5a eliciting YAP activation was pivotal for LNM-GCs reprogramming BM-MSCs. **a**–**d** Western blotting analysis of p-YAP^s127^ and YAP levels in BM-MSCs treated with GC exosomes (**a**), serum exosomes isolated from healthy individuals (H), GC patients without (NM), and those with regional LNM (M) (**b**), exosomes from lentivirus-infected AGS cells (**c**), and exosomes from oligonucleotide-transfected SGC-7901 cells (**d**). **e**–**j** BM-MSCs were pretreated with verteporfin before incubation with SGC-7901 exosomes (**e**, **g**, **h**) and serum exosomes isolated from GC patients with LNM (**f**, **i**, **j**). **e**, **f** Western blot analysis of CTGF and CYR61 in BM-MSCs. **g**, **i** Detection of α-SMA expression in BM-MSCs (magnification, ×200; scale bars, 50 μm). **h**, **j** Numbers of migrated and invaded GCs and formed tubule junctions. Data are presented as the mean ± SD of three independent experiments. Statistical significance was assessed using Student’s *t* test. ***P < 0.0001; **P < 0.01.

Yes-associated protein (YAP), a core transcriptional factor of the hippo pathway, plays an important role in maintaining the tumor-promoting role of primary GC tissue-derived MSC-like cells (GC-MSCs) [20]. YAP is also involved in modulating the transition of BM-MSCs into cancer-associated fibroblasts (CAFs) [21]. Wnt5a, as a noncanonical Wnt member, induces activation of YAP signaling, thereby contributing to malignant tumor progression. This alternative mechanism of Wnt signaling in GC has been garnering increased attention [22–24]. To investigate whether exosome-delivered Wnt5a educated BM-MSCs by activating YAP signaling, we measured the level of p-YAP and YAP in BM-MSCs treated with different exosomes. Compared with their corresponding controls, SGC-7901 derived exosomes, Wnt5a-enriched AGS exosomes, and serum exosomes isolated from GC patients with LNM stimulated YAP dephosphorylation in BM-MSCs (Fig. 6a–c). However, Wnt5a-deficient exosomes exerted the opposite effects, suppressing YAP expression (Fig. 6d). These results indicate that YAP activation may be involved in exosomal Wnt5a-mediated education of BM-MSCs.

To elucidate whether YAP activation is indispensable for exosomal Wnt5a-mediated BM-MSC reprogramming, verteporfin, a small-molecule inhibitor of YAP/TEAD binding, was used to pre-treat BM-MSCs before incubation with SGC-7901 exosomes and serum exosomes from GC patients with LNM. Connective tissue growth factor (CTGF) and cysteine-rich angiogenic inducer 61(Cyr61), the two downstream effectors of the Hippo signaling pathway, were significantly suppressed in BM-MSCs after Verteporfin treatment, reflecting inhibition of YAP/Hippo signaling by Verteporfin (Fig. 6e, f). In BM-MSCs, pretreatment with verteporfin suppressed a-SMA expression and tumor-promoting capacity, conferred by treatment with SGC-7901 exosomes (Fig. 6g, h). Similarly, verteporfin also attenuated the BM-MSC-educating effects of serum exosomes isolated from GC patients with LNM (Fig. 6i, j). These results imply that activation of YAP signaling elicited by exosomal Wnt5a is pivotal in LNM-GC-mediated reprogramming of BM-MSCs.

### Activation of YAP signaling is required for the maintenance of enhanced tumor-promoting phenotype and function in GLN-MSCs

**Fig. 7:**
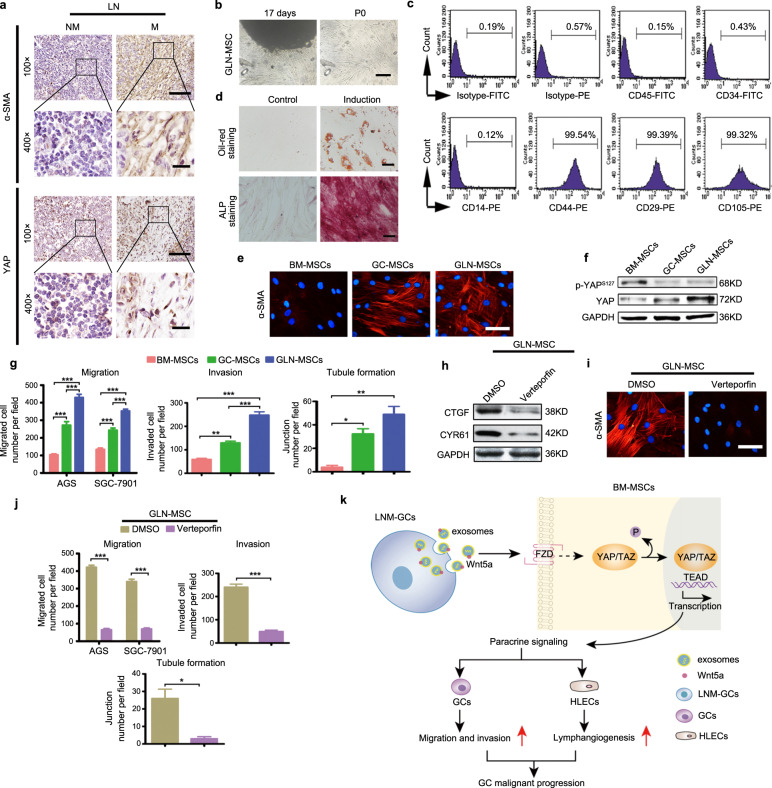
YAP signaling is critical for the maintenance of tumor-promoting phenotype and function in GLN-MSCs. **a** Immunohistochemical analysis of α-SMA and YAP expression in regional metastatic LN (M) and nonmetastatic LN (NM) tissues (magnification, ×100, scale bars, 100 μm; magnification, ×400; scale bars, 20 μm). **b**–**d** Isolation and characterization of GLN-MSCs. **b** Representative images of GLN-MSCs at day 17 and generation P0 in primary culture (magnification, ×100, scale bars, 200 μm). **c** MSC-associated surface markers detected by flow cytometry. **d** Analysis of adipogenic and osteogenic differentiation potentials (magnification, ×200, scale bars, 100 μm). **e**–**g** Comparative analysis of tumor-promoting phenotype, YAP-activation status, and function in BM-MSCs, GC-MSCs, and GLN-MSCs. **h**–**j** Influence of YAP-signaling inhibition with verteporfin on tumor-promoting phenotype and properties of GLN-MSCs. **h** Western blot analysis of CTGF and CYR61 in GLN-MSCs. **e**, **i** Detection of α-SMA expression in MSCs (magnification, ×200; scale bars, 50 μm); **f** Western blotting analysis of p-YAP^s127^ and YAP levels in MSCs. **g**, **j** Analysis of tumor-promoting properties in MSCs. **k** A graphic illustration of LNM-GCs educating BM-MSCs via exosomal Wnt5a-eliciting activation of YAP signaling. Data are presented as the mean ± SD of three independent experiments. Statistical significance was calculated using one-way ANOVA with post hoc Tukey’s test. ***P < 0.0001; **P < 0.01; *P < 0.05.

The results obtained in our present study show that LNM-GCs were specifically able to reprogram BM-MSCs into cancer-associated MSCs. Whether MSC-like cells exist in metastatic LN of GC patients remains unknown. Our results show that regional metastatic LN contained high numbers of α-SMA-positive stromal cells; however, this cell type was rarely observed in nonmetastatic LN. Distribution of α-SMA-positive stromal cells in metastatic LN showed that these spindle cells, localized at the same sites, were also positive for YAP immunolabeling, which was mainly expressed in the nucleus (Fig. 7a). We examined the morphology of these spindle stromal cells isolated from regional metastatic LN tissues, and observed that these cells showed BM-MSC-like morphological characteristics with a similar profile of surface marker expression (Fig. 7b, c). We performed staining on these spindle cells to detect their adipogenic and osteogenic differentiation potential, which identified their MSC-like multiple differentiation ability (Fig. 7d). Therefore, these cells were designated as MSC-like cells derived from regional metastatic LN tissues of GC (GLN-MSCs).

Previously, we had obtained GC-MSCs from primary GC tissues [25]. In our present study, we performed a comparative analysis of BM-MSCs, GC-MSCs, and GLN-MSCs (Fig. 7e–g). The expression levels of α-SMA in GLN-MSCs and GC-MSCs were similar, but both were higher than that in BM-MSCs (Fig. 7e). The levels of phosphorylated YAP were reduced in both of GLN-MSCs and GC-MSCs compared with those in BM-MSCs, but protein levels of YAP in GLN-MSCs were the highest among the three MSCs (Fig. 7f). The ability to promote GC migration and invasion, as well as HLEC tubule formation, was higher in GLN-MSCs and GC-MSCs relative to those of BM-MSCs; however, GLN-MSCs showed stronger tumor-promoting potential than that of GC-MSCs (Fig. 7g). Treatment with verteporfin inhibited YAP signaling, markedly reduced a-SMA expression in GLN-MSCs and suppressed their tumor-promoting capacity (Fig. 7h–j). These results suggest that MSC-like stromal cells are indeed present in regional metastatic LN of GC patients, and that these cells display a typical tumor-promoting phenotype and strong oncogenic capacity. Our results also indicate that YAP activation was necessary for the maintenance of tumor-promoting phenotype and function of GLN-MSCs.

## Discussion

LNM, a typical malignant behavior of GCs, directly determines therapeutic response and prognosis in GC patients. The complicated LNM process is associated with enhanced migration and invasion in GCs cells, and with lymphangiogenesis in primary sites [26]. Recent studies have shown the importance of the pre-metastatic niche in LNM [27]. The molecular mechanisms underlying occurrence and progression of LNM in GC remain unclear. Our present study shows that LNM-GCs specifically reprogrammed BM-MSCs to promote GC migration and invasion and that they also promoted lymphangiogenesis via activation of YAP signaling by exosomal Wnt5a. These findings provide new insights into the mechanism of LNM from the perspective of the tumor microenvironment.

BM-MSCs possess tumor-tropic characteristics and incorporate into the tumor stroma to promote progression of malignant tumors. Interactions between MSCs and cancer cells are key drivers in this process [7, 28], and highly malignant cancer cells display an enhanced ability to educate BM-MSCs [9–11]. Similar to the results obtained in previous studies, our results show that LNM-GCs, but not primary GCs, could reprogram BM-MSCs. Increased migratory and invasive capacity in GCs, as well as lymphangiongenesis, are important factors that contribute to LNM. Herein, we show that LNM-GC-educated BM-MSC-CM, induced lymphangiogenesis, and promoted LNM-GC and primary GC migration. An in vivo study showed that the educated BM-MSCs not only prompted further metastasis of LNM-GCs, but also promoted LNM of primary GCs. Our results, obtained using acquisition and functional analysis of GLN-MSCs, further support this notion. These findings provide new insights into BM-MSC-mediated triggering of LNM and induction of persistent LNM metastasis in GC.

Exosomes, secreted by tumor cells in melanoma, lymphoma, and lung cancer, endow MSCs with tumor-favorable phenotype [29, 30]. Similarly, different fractions separated from LNM-GC-CM confirmed that exosomes were the key fraction regulating LNM-GC-mediated education of BM-MSCs. Recently, Shen et al. reported that AGS-derived exosomes affect the immune regulatory function of MSCs, which may promote GC progression by sustaining an inflammatory microenvironment [31]. This finding appears to contradict our results; however, it is possible that AGS exosomes endow BM-MSCs only with tumor-favorable immunoregulatory function, but not with the ability to directly enhance GC migration, invasion, and lymphangiogenesis. The regulatory role of exosomes is mediated by their cargo molecules. Interestingly, lung cancer cells can induce a pro-inflammatory phenotype in MSC via exosomal surface HSP70 [30]. Osteosarcoma extracellular vesicles can educate MSCs via membrane-associated TGFβ [32]. Consistently, our present study also shows that surface proteins were key in mediating the reprogramming of BM-MSCs by LNM-GC exosomes. In contrast to the findings obtained in previous studies [30, 32], we identified Wnt5a is a different surface protein involved in MSC reprogramming. These discrepancies may be due to the different characteristics of MSCs derived from different tissues, and differences in tumor cell-mediated education of MSCs. This notion also explains the complexity of MSC programming by tumor cells, and future studies should comprehensively investigate the various related surface proteins involved in this process.

One recent work showed that targeting Wnt signaling was effective in primary GC and suggested that this approach could also work in metastatic GC [33]. Our own findings confirmed that inhibition of Wnt5a and its downstream signaling suppressed GC metastasis by blocking the educative effect of BM-MSC. In fact, wnt5a is acknowledged as a GC metastasis-associated gene by increasing metastatic potential of GCs [34]. One recent meta-analysis concluded that Wnt5a expression was positively correlated with several clinicopathological parameters of GC, including LNM, tumor invasion depth, and advanced stages [19]. Only LNM-GCs responded to siRNA or specific antibody against Wnt5a but primary GCs did not [35]. Wnt5a was highly expressed in LNM-GCs and crucial to their education of BM-MSCs, and primary GCs had lower endogenous levels of Wnt5a, which explains the differences in the response to Wnt5a antibody and siRNAs between LNM-GCs and primary GCs. The expression and functional complexity of Wnt5a in tumors also received considerable attention. In particular, a Wnt5a mimicking peptide, Foxy-5, has been shown to be a promising agent to suppress metastasis of patients with prostate cancer or breast cancer with low endogenous expression of Wnt5a, but it did not affect those with higher endogenous levels of Wnt5a [36, 37]. We found that primary GCs had low endogenous levels of Wnt5a relative to LNM-GCs. However, Wnt5a overexpression in primary GCs did not suppress GC metastasis. Rather, it increased their ability to educate BM-MSCs. LNM-GCs reprogramed BM-MSC depending on exosomal Wnt5a. We infer that up-regulation of Wnt5a maybe not a suitable therapeutic strategy for GC metastasis.

Increased protein levels of Wnt5a were not only detected in LNM-GCs and their exosomes, but also in serum exosomes derived from GC patients with regional LNM. Based on the role of exosomal Wnt5a in education of BM-MSCs by LNM-GCs, this finding partially explains why only the serum exosomes obtained from GC patients with LNM could reprogram BM-MSCs. Our results, obtained using an ELISA immunoassay in a mouse model of LNM, indicate that LNM-GCs selectively secreted exosomes into the circulation, and that exosomal Wnt5a should be explored as a potential indicator in the monitoring of GC with LNM. Previously, Błogowski et al. have shown increased numbers of circulating BM-MSCs in the peripheral blood of GC patients [38]. Combined with our results showing that serum exosomes derived from GC patients with regional LNM could educate BM-MSCs, these findings suggest that LNM-GCs may secrete exosomes to remotely educate circulating BM-MSCs and to recruit BM-MSCs to LN for reprogramming.

YAP, a crucial effector of the hippo signaling pathway, is activated by dephosphorylation to enter into the nucleus, where it interacts with TEAD to transcriptionally regulate the expression of downstream genes [39]. YAP is required in GC-MSC-mediated tumor promotion [20] and in regulation of the transition of BM-MSCs into CAFs [21]. Wnt5a potentially activates YAP/TAZ to elicit alternative Wnt signaling in bone marrow stromal cells [22], and even exosome-carried Wnt5a can activate YAP signaling [40]. Consistently, we revealed that LNM-GC-mediated education of BM-MSCs occurred via activation of YAP signaling by exosomal Wnt5a. This finding was confirmed by our results showing positive YAP immunolabeling in the nuclei of resident stromal cells in metastatic LN tissues of GC patients. We also show that YAP signaling activation was required for sustaining the tumor-favorable phenotype and function of GLN-MSCs. GC-MSCs and GLN-MSCs, characterized as important stromal cells in GC, display tumor-promoting phenotype and function; however, the differences between these two cell types are yet to be elucidated. We speculated that YAP activation is common in GC-associated MSCs, and that the degree of YAP activation is determined by the status of GC malignancy. YAP should be explored as a promising therapeutic target in suppression of GC development via blocking of BM-MSC education. YAP expression should also be evaluated in cancer-associated MSCs to determine whether its expression levels reflect the malignant progression of GC. Although GC-MSCs isolated from primary GC tissues can promote GC migration and invasion, we cannot assume that BM-MSCs can be reprogrammed under primary conditions. These primary GC tissues were obtained from GC patients with LNM in the present study. We have found that not all GC-MSCs established by our research group have tumor-promotive functions, but all of the obtained GLN-MSCs have. The oncogenic ability of GC-MSCs is associated with LNM setting, which further supports the conclusion that LNM-GCs can reprogram BM-MSCs.

Summarily, our study indicates that LNM-GCs specifically educated BM-MSCs by activating YAP signaling via exosome delivery of Wnt5a (Fig. 7k). The results obtained in our study revealed three main differences between LNM-GCs and primary GCs from the perspective of BM-MSC reprogramming: 1. The ability to reprogram BM-MSCs; 2. the endogenous expression levels of Wnt5a in cells and their exosomes; and 3. the ability to activate YAP signaling in MSCs. These findings provide new insights into the mechanism underlying LNM in GC and may also identify potential therapeutic targets and detection indicators for GC patients with LNM.

## Materials and methods

Details regarding cell biology, molecular biology, reagents, and statistical analyses can be found in the Supplementary Information.

## Supplementary information

Supplementary information

Supplementary Table 1

Supplementary Figure 1

Supplementary Figure 2

Supplementary Figure 3

Supplementary Figure 4
