# Supplementary material for: Lymph node metastasis-derived gastric cancer cells educate bone marrow-derived mesenchymal stem cells via YAP signaling activation by exosomal Wnt5a
Source: Oncogene. 2021 Mar 2;40(12):2296–308. doi: 10.1038/s41388-021-01722-8 (PMC7994201; doi:10.1038/s41388-021-01722-8)
Supplement: Supplementary file 1 — Supplementary information [file 41388_2021_1722_MOESM1_ESM.pdf]

Supplementary information summary

1. **Supplementary Material and Methods**
2. **Supplementary references**
3. **Supplementary Table 1** (PDF)

   Sequences of siRNAs against Wnt5a
4. **Supplementary Figure 1** (PDF)

   Exosome characterization by NTA and Western blot analyses
5. **Supplementary Figure 2** (PDF)

   LNM-GC-educated BM-MSCs enable primary GCs to acquire the ability to metastasize to LNs
6. **Supplementary Figure 3** (PDF)

   Removal of proteins from HGC-27 exosomes suppresses their role in BM-MSC education
7. **Supplementary Figure 4** (PDF)

   Wnt5a is highly expressed in LNM-GCs and enriched in their exosomes, and knockdown of exosomal Wnt5a attenuates the role of HGC-27 cells in BM-MSCs reprogramming

Supplementary Materials and Methods

Cells and clinical samples

Human GC cell lines (AGS, MGC-803, SGC-7901 and HGC-27) and lymphatic capillary endothelial cells (HLECs) were obtained from the Shanghai Cell Bank of the Chinese Academy of Sciences (Shanghai, China) and cultured in Dulbecco's Modified Eagle Medium (DMEM) (Gibco, Thermo Fisher Scientific, Inc., MA, USA) containing 10% fetal bovine serum (FBS) (Gibco) at 37°C in humidified air with 5% CO_2. Bone marrow-derived mesenchymal stem cells (BM-MSCs) and primary GC tissue-derived MSC-like cells (GC-MSCs) were isolated, characterized, and maintained as described previously [1]. MSC-like cells from metastatic LN tissues of patients with GC (GLN-MSCs) were isolated and characterized using the same methods described previously [1, 2]. All these cell lines have been recently tested negative for mycoplasma contamination. The media of GCs and MSCs were separately harvested when cell confluence was at 80%. These media were subjected to centrifugation (1500 rpm for 10min), and filtration through a 0.22μm membrane (Millipore, Germany). The final media were designated as conditioned media (CM). Clinical tissues and sera were collected at the Affiliated Peoples' Hospital of Jiangsu University and Jiangsu Cancer Hospital. Signed informed consent was obtained from all participants. The protocols used in this study were approved by the Ethics Committee of Jiangsu University and Jiangsu Cancer Hospital, and conducted in accordance with the Helsinki Declaration.

Separation and characterization of exosomes

GC-CM were ultra-centrifuged at 110,000 g for two hours. The upper supernatant contained the exosome-free fraction. The pellet, which was washed with phosphate buffered saline (PBS) and ultra-centrifuged at 110,000 g for 30 min, contained the exosome fraction. The pooled serum was prepared by mixing 40μl of each serum from five people in each group. Serum exosomes were isolated using ExoQuick exosome precipitation solution (SBI System Biosciences, CA, USA) as described previously [3]. Exosomes were characterized using transmission electron microscopy (TEM), nanoparticle tracking analysis (NTA), and western blotting as described previously [3]. CD81 expression was analyzed using Gallios flow cytometry (Beckman, CA, USA) with an Exo-Flow capture kit (SBI) according to the manufacturer's instructions.

Cell transfection and lentivirus infection

Small interfering RNAs (siRNAs) against Wnt5a and corresponding negative-control oligonucleotide (NC) were purchased from Genepharma (Shanghai, China). Each oligonucleotide was transfected into LNM-GCs at the final concentration of 50 nM using Lipofectamine 2000 (Invitrogen, Thermo Fisher Scientific, Inc., MA, USA). The sequences of all oligonucleotides are listed in Supplementary Table 1. WNT5A (NM_003392) Human Tagged ORF Clone and pCMV6-AC-GFP mammalian expression vector were obtained from OriGene Technologies, Inc. (MD, USA). AGS cells were transfected with the vectors for 48h. They were then treated with G418 (600 mg/ml) to screen the resistant cells and finally maintained at a lower concentration of G418 (400 mg/ml). Wnt5a-overexpressing lentivirus and control virus were purchased from Genechem (Shanghai, China). AGS cells were infected with the lentivirus at the multiplicity of infection (MOI) of 20. Polybrene (at the final concentration of 50 μg/ml, Genechem) was used to improve the efficiency of infection.

Treatment of MSCs with CM, exosomes, verteporfin and cycloheximide

BM-MSCs were seeded in six-well plates at the density of 5×10^4 per well and allowed to attach overnight. BM-MSCs were treated with 2 ml GC-CM premixed with an equal volume of refreshed BM-MSC culture medium, or with 100 μg/ml exosomes for 48 h. BM-MSCs were pretreated for 24 h with 200 ng/ml verteporfin (MedChemExpress, Monmouth Junction, NJ, USA) in DMSO, and then treated with GC-CM or exosomes for 48 h. GLN-MSCs were seeded in six-well plates at the density of 5×10^4 cells per well, allowed to attach overnight, and then treated with 200 ng/ml verteporfin for 24 h. Then, MSC culture media were replaced with fresh media, and the cells were further cultured for 24 h. The culture media were collected and used for preparation of MSC-CM. MSCs were collected for protein detection by immunofluorescence assay and Western blot. BM-MSCs were planted in six-well plates at the density of 5×10^4 per well and attached overnight. They were treated with 15 μg/ml Cycloheximide (CHX) (Beyotime Biotechnology, Shanghai, China) alone or together with exosomes, and lysed at the indicated times for Western blot analysis.

Exosome internalization and immunofluorescence assay

BM-MSCs were seeded in 24-well plates at the density of 1.5×10^4 per well and allowed to attach overnight. Cells were starved for 4h, then treated with 10 μg/ml exosomes for 6 h and fixed

with 4% paraformaldehyde. After fixation, cells were permeabilized with 0.1% Triton X-100 for 10 min, blocked with 5% bovine serum albumin (BSA) for 20 min, and then stained with YF®555- phalloidin (US Everbright® Inc., Suzhou, China) for 45 min and Hoechst 33258 (Boster Biological Technology, Wuhan, China) for 5 min. Immunofluorescence labeling of alpha-smooth muscle actin (α-SMA) in MSCs was performed as described previously [4, 5]. Primary antibody against α-SMA (BM0002) were purchased from Boster Biological Technology. Uptake of exosomes by MSCs and α-SMA expression in MSCs were visualized by fluorescence microscopy in at least five fields (Zeiss AG, Oberkochen, Germany).

Migration and invasion assay

For the migration assay, 1×10^5 AGS and 8×10^4 SGC-7901, separately suspended in serum-free media, were added into the upper chamber and incubated for 10 h; the media in the bottom chamber contained 10% FBS, used as a chemotactic agent. For the invasion assay, 50 μl matrigel (BD Bioscience, San Jose, CA, USA), diluted at 3:1 using serum-free media, was added into the upper chamber, and the cells were incubated at 37°C for 30 min. The media containing 10% FBS were added into the lower chamber, while 1×10^5 HGC-27, suspended in serum-free media, were placed into the upper chamber and incubated at 37°C for 24 h. The migrated and invaded cells were stained with crystal violet, and then viewed and counted in six fields.

Tubule-formation analysis

Matrigel (50 μl, BD Bioscience) was added into each well of each 96-well plate and incubated at 37° C for 30 min. HLECs were digested using trypsin and suspended in serum-free DMEM at the concentration of 4×10^4 cells/ml. This cell suspension was pre-mixed with an equal volume of MSC-CM. Then, 100 μl mixed cell suspension was added into each well of a 96-well plate containing matrigel and incubated at 37°C for 8 h. Tubule formation was observed under a microscope and analyzed using Image J software.

Western blotting

Cellular and exosomal proteins were extracted and detected by western blotting as described previously [3]. Primary antibodies against CD9 (BS60359), CD63 (BS72936), GAPDH (MB9231, Bioworld Technology, Inc., MN, USA), phospho-YAP1-S127(AP0489), YAP1(A1001), CTGF(A11067), CYR61 (A1111, ABclonal, Inc., Wuhan, China), Wnt5a (sc-365370, Santa Cruz

Biotechnology, Inc., TX, USA) and mouse monoclonal turboGFP antibody (TA150041, OriGene) were used according to manufacturers' instructions. The secondary antibodies, HRP goat anti-rabbit IgG (AS014) and mouse IgG (AS003, ABclonal) were also used per the manufacturer's instructions.

Serum exosomal Wnt5a detection by ELISA

Exosomes were isolated from 200 μl serum, lysed in RIPA buffer, and analyzed using an ELISA immunoassay. Two different human antibodies against Wnt-5a (MAB6452 and MAB6451, R&D Systems, Inc., MN, USA) were used as the capture and coating antibody. DuoSet ELISA ancillary reagent Kit 2 (R&D Systems) with biotin-conjugated goat anti-mouse IgG(H+L) (SA00004-1) and HRP-conjugated streptavidin (SA00001-0, Proteintech Group, Inc., Rosemont, IL, USA) were used for the detection of serum exosomal Wnt5a. All the reagents were used at the concentrations recommended by the manufacturers. Recombinant human/mouse Wnt5a protein (R&D Systems) was used to construct a standard curve.

Immunohistochemistry (IHC)

Instant SABC-POD Kit (Boster Biological Technology) was used as described previously [3]. Primary antibodies against α-SMA (BM0002, Boster Biological Technology), YAP1 (A1001, ABclonal), and pan cytokeratin [AE1/AE3] (ab27988, Abcam, Cambridge, UK) were used for target-protein detection. The expression and distribution of α-SMA, YAP, and pan cytokeratin-positive cells in LN tissues were analyzed using a microscope.

LNM model

Male BALB/c nude mice (3-4 weeks old) were purchased from the Model Animal Research Center of Nanjing University (Nanjing, China) and were randomly used for LNM model establishment. SGC-7901 cells or AGS cells were treated with BM-MSC-CM, collected, and suspended in PBS at a concentration of 1×10^7 cells/ml. Then, 200 μl cell suspension was injected into the left footpad of each mouse. The mice of SGC-7901 tumor model were sacrificed 3~4 weeks later, and those of AGS tumor model were sacrificed 10 weeks later. Three mice in each group were used for evaluating the educative effect of LNM-GC exosome. Five mice in each group were used to determine the role of exosomal Wnt5a in BM-MSC education. The draining popliteal LNs were blindly harvested, imaged, weighed, and assessed using immunohistochemical

analysis. All procedures involving animals were approved by the Committee on the Use and Care of Animals of Jiangsu University, and were conducted in accord with the guidelines of Jiangsu University.

Statistical analyses

GraphPad Prism 5 software was used for statistical analysis. Sample sizes in each experiment and animal model were determined based on our previous studies [2,4,5,6]. All the data were obtained from three independent experiments. The samples with values more than twice the SEM of the mean were excluded. Data are presented as means ± SD. The variance is similar between the groups that were being statistically compared. The data met the assumptions of the tests. Statistical significance was calculated using Student's *t* test or one-way analysis of variance (ANOVA) with Tukey's post hoc test. P <0.05 indicates statistical significance.

Supplementary references

1. Cao H, Xu W, Qian H, Zhu W, Yan Y, Zhou H *et al*. Mesenchymal stem cell-like cells derived from human gastric cancer tissues. *Cancer Lett* 2009; **274**: 61-71.

2. Wang M, Chen B, Sun XX, Zhao XD, Zhao YY, Sun L *et al*. Gastric cancer tissue-derived mesenchymal stem cells impact peripheral blood mononuclear cells via disruption of Treg/Th17 balance to promote gastric cancer progression. *Exp Cell Res* 2017; **361**: 19-29.

3. Wang M, Zhang H, Yang F, Qiu R, Zhao X, Gong Z *et al*. miR-188-5p suppresses cellular proliferation and migration via IL6ST: A potential noninvasive diagnostic biomarker for breast cancer. *J Cell Physiol* 2020; **235**: 4890-4901.

4. Zhu M, Wang M, Yang F, Tian Y, Cai J, Yang H, *et al*. miR-155-5p inhibition promotes the transition of bone marrow mesenchymal stem cells to gastric cancer tissue derived MSC-like cells via NF-kappaB p65 activation. *Oncotarget* 2016;7:16567-16580.

5. Wang M, Yang F, Qiu R, Zhu M, Zhang H, Xu W *et al*. The role of mmu-miR-155-5p-NF-kappaB signaling in the education of bone marrow-derived mesenchymal stem cells by gastric cancer cells. *Cancer Med* 2018; **7**: 856-868.

6. Wang M, Zhao C, Shi H, Zhang B, Zhang L, Zhang X *et al*. Deregulated microRNAs in gastric cancer tissue-derived mesenchymal stem cells: novel biomarkers and a mechanism for gastric cancer. *Br J Cancer* 2014; **110**: 1199-1210.

Supplementary Table 1 Sequences of siRNAs against Wnt5a

name	Sequences (5'-3')
Negative control（NC）	Sense: UUCUCCGAACGUGUCACGUTT
	Antisense: ACGUGACACGUUCGGAGAATT
siRNA-1	Sense: GUGGUCGCUAGGUAUGAAUTT
	Antisense: AUUCAUACCUAGCGACCACTT
siRNA-2	Sense: CGCGAAGACAGGCAUCAAATT
	Antisense: UUUGAUGCCUGUCUUCGCGTT
siRNA-3	Sense: GCUACGUCAAGUGCAAGAATT
	Antisense: UUCUUGCACUUGACGUAGCTT

Supplementary Figure 1 Exosome characterization by NTA and Western blot analyses

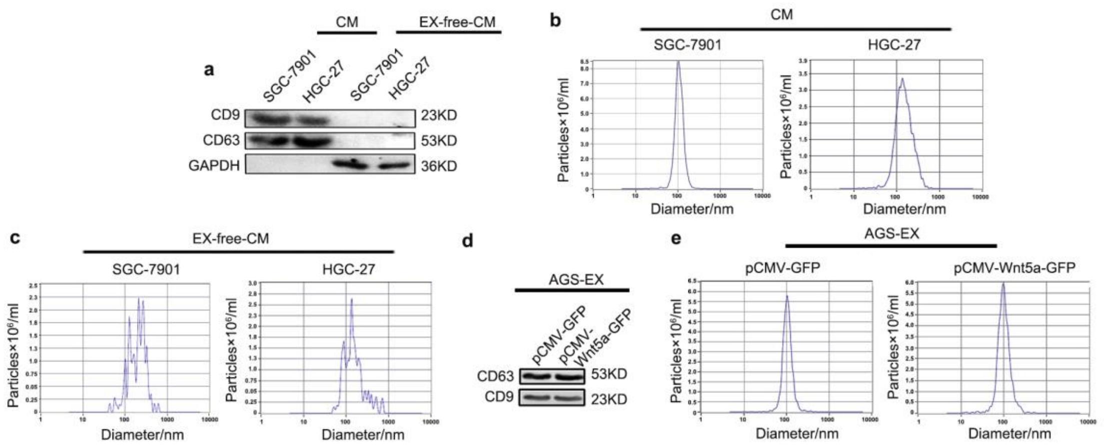

a-c, exosome detection in GC-CM and exosome-free-CM. d-e, characterization of exosomes derived from AGS stably transfected with pCMV-GFP and pMCV-Wnt5a-GFP. a and d, exosomal marker CD63 and CD9 detection by Western blot. b, c and e, NTA analysis.

Supplementary Figure 2 LNM-GC-educated BM-MSCs enable primary GCs to acquire the ability to metastasize to LN

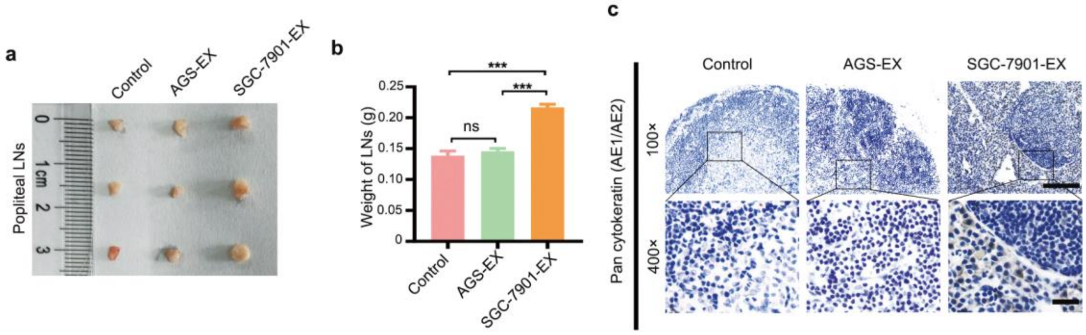

a, The draining popliteal LNs from each group were harvested and imaged. b, Weight of popliteal LNs. c, Immunohistochemical analysis of pan cytokeratin (AE1/AE3) expression in popliteal LNs. Representative images from each group are shown (magnification, ×100, scale bars, 100 μm; magnification, ×400, scale bars, 20 μm;). Data are presented as the mean ± SD of three independent experiments. Statistical significance was assessed using one-way ANOVA followed by Tukey's test. ***, *P*<0.0001; ns, non-significant.

Supplementary Figure 3 Removal of proteins from HGC-27 exosomes suppresses their role in BM-MSC education

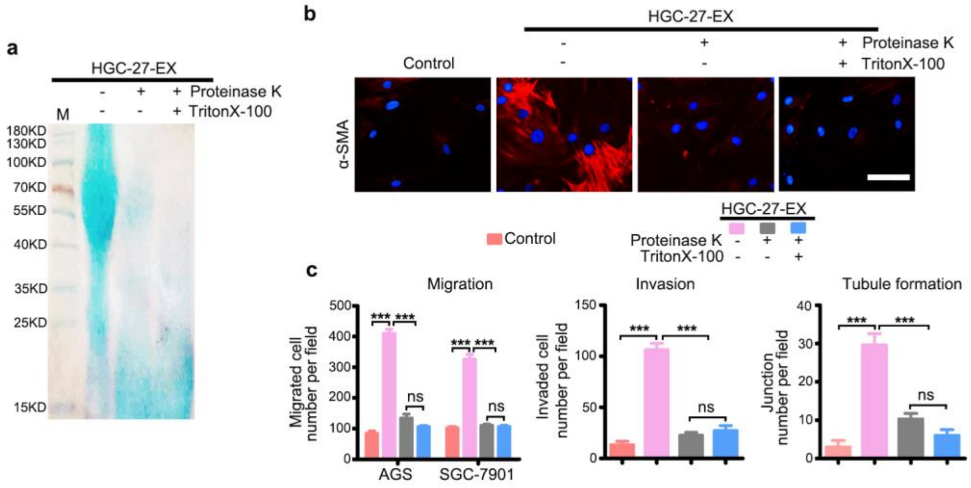

a, SDS-PAGE and Coomassie brilliant blue stain were used to confirm that proteins were indeed removed from HGC-27 exosomes. b-c, Protein removal suppressed the effect of HGC-27 exosomes educating BM-MSCs. b, Immunofluorescence detection of α-SMA expression in BM-MSCs (magnification, ×200; scale bars, 50 μm). c, Analysis of tumor-promoting properties in BM-MSCs. Quantification of migrated and invaded GCs, and formed tubule junction. Data are presented as the mean ± SD of three independent experiments. Statistical significance was assessed using one-way ANOVA followed by Tukey's test. ***, P<0.0001; ns, non-significant.

Supplementary Figure 4 Wnt5a is highly expressed in LNM-GCs and enriched in their exosomes, and knockdown of exosomal Wnt5a attenuates the role of HGC-27 cells in BM-MSCs reprogramming

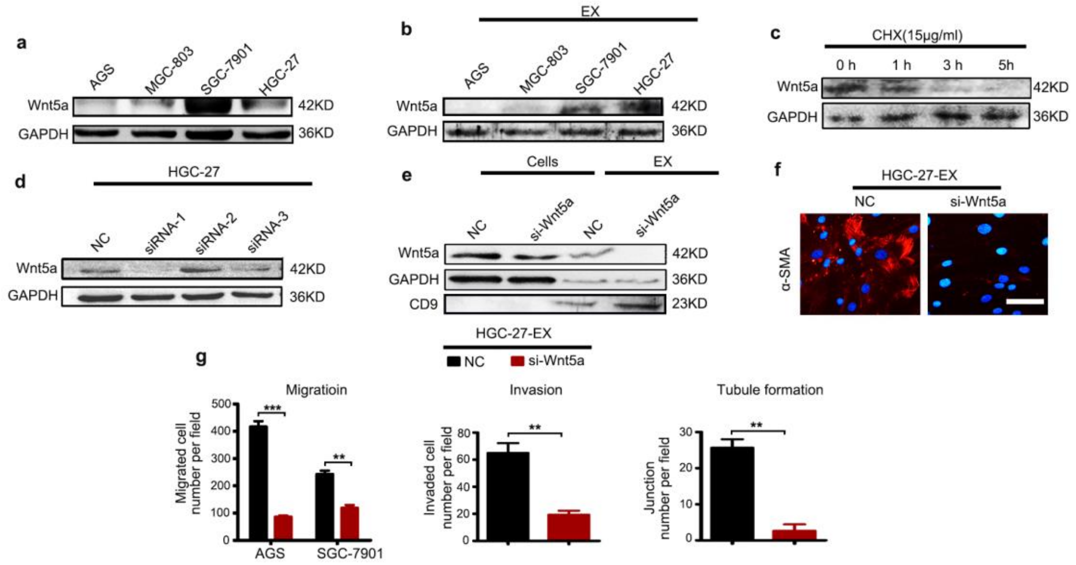

a and b, Western blot analysis of Wnt5a in GCs (a) and their exosomes (b). c, Wnt5a protein detection in BM-MSCs after CHX treatment at the indicated time. d, Screening for si-Wnt5a in HGC-27. NC was used as the control. e, Western blot analysis of Wnt5a expression in HGC-27 cells, and analysis of their secreted exosomes after transfection of HGC-27 with si-Wnt5a and NC. e-g, si-Wnt5a- and NC- transfected HGC-27 exosomes were separately used to treat BM-MSCs in order to compare their effects with respect to education of BM-MSCs. f, Immunofluorescence assay of α-SMA expression in BM-MSCs (magnification, ×200; scale bars, 50 μm). g, Analysis of tumor-promoting properties of BM-MSCs. Quantification of migrated and invaded GCs and formed tubule junctions. Data are presented as the mean ± SD of three independent experiments. Statistical significance was assessed using Student's *t* test. ***, $P<0.0001$; **, $P<0.01$.
