# Supplementary material for: Lymph node metastasis-derived gastric cancer cells educate bone marrow-derived mesenchymal stem cells via YAP signaling activation by exosomal Wnt5a
Source: Oncogene. 2021 Mar 2;40(12):2296–308. doi: 10.1038/s41388-021-01722-8 (PMC7994201; doi:10.1038/s41388-021-01722-8)
Supplement: Supplementary file 2 — Supplementary Table 1 [file 41388_2021_1722_MOESM2_ESM.pdf]

Supplementary Table 1 Sequences of siRNAs against Wnt5a

name	Sequences (5'-3')
Negative control（NC）	Sense: UUCUCCGAACGUGUCACGUTT
	Antisense: ACGUGACACGUUCGGAGAATT
siRNA-1	Sense: GUGGUCGCUAGGUAUGAAUTT
	Antisense: AUUCAUACCUAGCGACCACTT
siRNA-2	Sense: CGCGAAGACAGGCAUCAAATT
	Antisense: UUUGAUGCCUGUCUUCGCGTT
siRNA-3	Sense: GCUACGUCAAGUGCAAGAATT
	Antisense: UUCUUGCACUUGACGUAGCTT
