# Supplementary figures and images for: Lymph node metastasis-derived gastric cancer cells educate bone marrow-derived mesenchymal stem cells via YAP signaling activation by exosomal Wnt5a
Source: Oncogene. 2021 Mar 2;40(12):2296–308. doi: 10.1038/s41388-021-01722-8 (PMC7994201; doi:10.1038/s41388-021-01722-8)

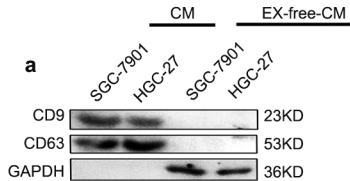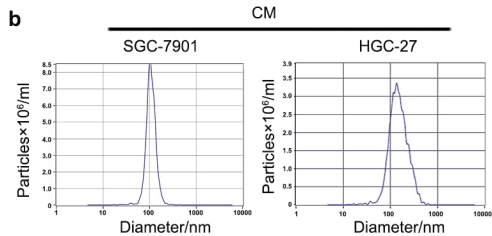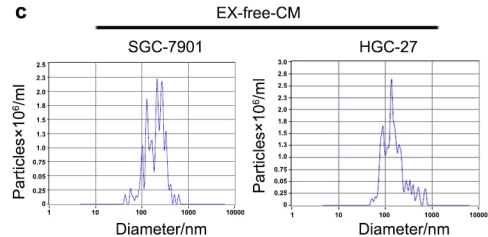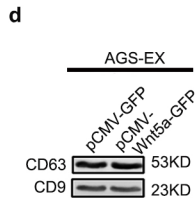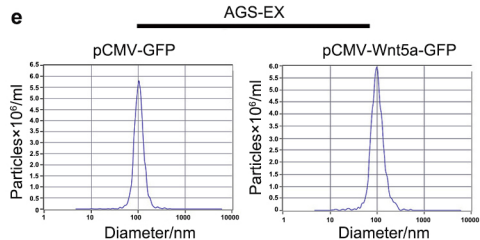

Supplement: Supplementary file 3 — Supplementary Figure 1 [file 41388_2021_1722_MOESM3_ESM.pdf]

**a**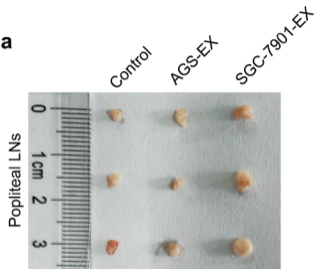**b**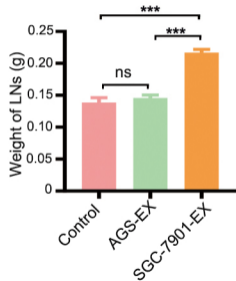**c**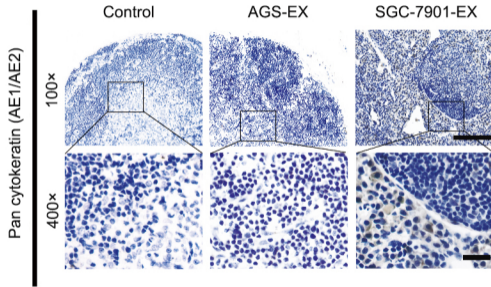

Supplement: Supplementary file 4 — Supplementary Figure 2 [file 41388_2021_1722_MOESM4_ESM.pdf]

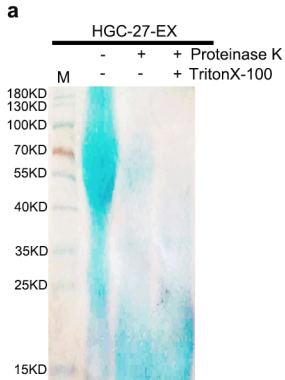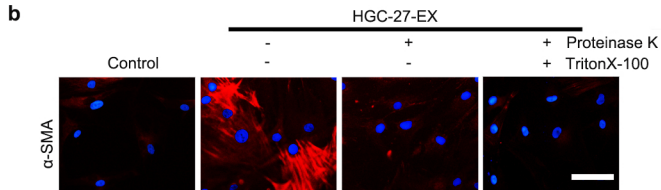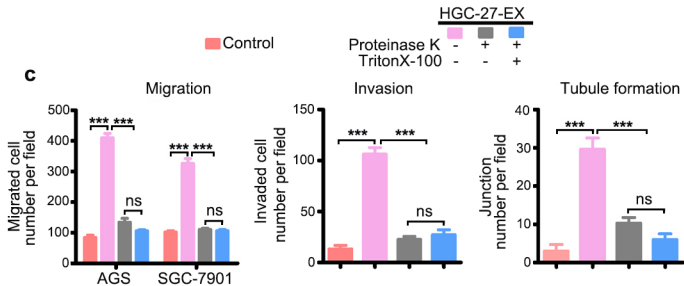

Supplement: Supplementary file 5 — Supplementary Figure 3 [file 41388_2021_1722_MOESM5_ESM.pdf]

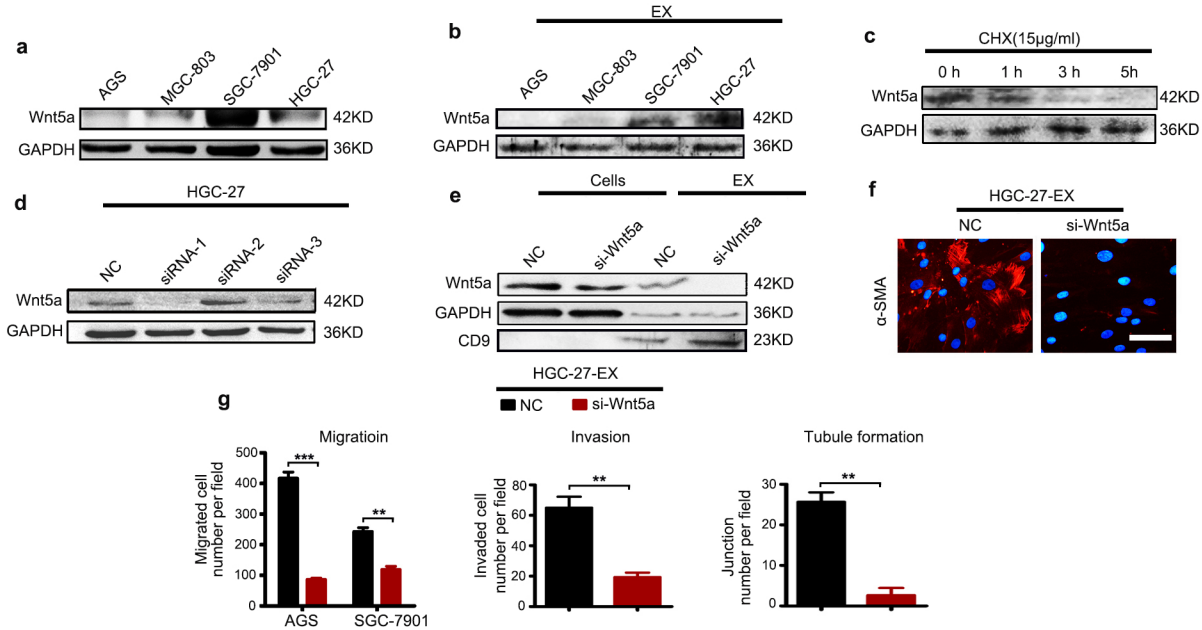

Supplement: Supplementary file 6 — Supplementary Figure 4 [file 41388_2021_1722_MOESM6_ESM.pdf]
